# Acute Rheumatic Fever in a COVID-19-Positive Pediatric Patient

**DOI:** 10.1155/2021/6655330

**Published:** 2021-04-12

**Authors:** Christa I. DeVette, Cherise S. Ali, Devon W. Hahn, Stephanie D. DeLeon

**Affiliations:** Department of Pediatrics, University of Oklahoma Health Sciences Center, Oklahoma City, OK, USA

## Abstract

Bacterial coinfection and COVID-19 have been reported in pediatric populations. We describe a case of Sydenham's chorea, which is exceedingly rare in developed countries, with concurrent COVID-19. Discussed here is the clinical course of an 8-year-old COVID-positive female with pure Sydenham's chorea and subclinical carditis from acute rheumatic fever. To our knowledge, there are no documented reports of acute rheumatic fever in a pediatric patient with coexisting COVID-19 infection.

## 1. Introduction

In December 2019, *SARS-CoV-2*, a coronavirus now commonly known as COVID-19, infected a small cohort of patients in China. Since then, COVID-19 has spread to roughly 31 million people with over 965,000 deaths globally. Most patients with COVID-19 exhibit mild flu-like symptoms; however, roughly 20% of patients are deemed critically ill with a subset of patients proceeding to septic shock and multiorgan failure [1]. In pediatric populations, multisystem inflammatory syndrome in children (MIS-C) is a rare but severe manifestation of COVID-19 that presents as shock with cardiac and gastrointestinal symptoms [2]. The presence of bacterial superinfection in severely ill COVID-19 patients has also been documented, with many patients requiring additional treatment for bacterial pneumonia or sepsis [3]. While we continue to rapidly learn about the impact of host genetic factors and age on the risks with infection, the epidemiology to date reveals a wide variation in disease course among confirmed cases, necessitating further investigation of disease manifestation and coexisting bacterial infection.

We report a pediatric patient presenting with isolated choreiform movements who was ultimately diagnosed with acute rheumatic fever (ARF) and COVID-19. ARF is a systemic disease associated with *Streptococcus pyogenes* (GAS) infection, which manifests with choreiform movements, migrating arthralgias, skin findings, and, importantly, cardiac involvement; these findings are summarized in the revised JONES diagnostic criteria [4]. Although ARF and its neurologic manifestations have been extensively studied, the pathophysiological consequences of COVID-19 coinfection remain unexplored.

## 2. Case Presentation

An 8-year-old previously healthy female presented with a 6-day history of involuntary twitching movements of her left arm. The patient's mother first noticed abnormal “quick jerks” of the left arm that progressed to involve the left leg and the right arm. Two days prior to presentation, her movements acutely worsened, and she developed lip smacking and intermittent grimacing. The patient's mother also reports that she had noticed generalized weakness, loss of coordination, and abnormal behavior including bouts of confusion, altered speech, random episodes of laughter, and difficulty sleeping secondary to movements in the last 48 hours. She denied any history of rash, sore throat, arthralgias, fever, or respiratory symptoms. Her parents had both tested positive for COVID-19 two weeks prior to her presentation; the patient and her siblings were not tested as they remained asymptomatic.

On physical examination, the patient was developmentally appropriate for her age and was overall well-appearing. She was awake, alert, and oriented. Her speech while answering questions was garbled by automatisms of her lips and face. She had prominent persistent choreiform movements in the right arm and right leg. She occasionally smacked her lips and grimaced. Her gait was altered due to movements in the right leg, and she required assistance climbing on and off the exam table. She exhibited nonspecific weakness while ambulating despite the ability to move all extremities against resistance during her exam. Her cardiac exam revealed regular rate and rhythm without a 1/6 systolic flow murmur heard at the left sternal border. She did not have dermatologic changes, lymphadenopathy, or pharyngeal abnormalities. The patient had normal affect throughout the exam. Her vitals were normal for her age.

Her initial evaluation included a positive rapid streptococcus swab and positive COVID-19 nasopharyngeal PCR. Her labs showed elevated titers of anti-streptolysin-O (455 IU/mL) and anti-DNAse-B (807 U/mL). She also had mildly elevated TSH of 7.2 mIU/L with normal free T4 and an ESR of 14 mm/hr. Transthoracic echocardiogram revealed mitral valve thickening and moderate mitral valve insufficiency, and her EKG was unremarkable. Given her extensive weakness, gait abnormality, and behavioral changes in the setting of positive COVID status, additional imaging was warranted due to clotting risk. Brain CT, MRI, and MRA were unremarkable for any acute processes. Notably, her MRI and MRA required sedation due to choreiform movements, which was complicated by her COVID-19 status and aerosolization risk.

The patient received intramuscular penicillin G as streptococcal treatment. She was also started on aspirin due to cardiac involvement and valproate twice daily for chorea. Clinically, she responded well to the valproate, and her movements had markedly decreased upon discharge. She remained COVID-19 positive on retesting two weeks following her initial test. Based on current CDC retesting guidelines, in the absence of COVID-19 symptoms, the patient did not receive further COVID-19 PCR testing at any outpatient follow-up appointments. At our patient's one-month follow-up, she endorsed continued fatigue and left arm choreiform movements that limited her ability to use her hand requiring escalation of her valproate. Her rheumatic heart disease (RHD) remained unchanged on follow-up echocardiogram outpatient. She is currently undergoing further workup for her chronic fatigue as this may also be due to COVID-19.

Finally, the patient was placed on monthly injections of penicillin for the next 10 years. All family members were tested for active streptococcal infection and treated accordingly to minimize the risk of reinfection and further cardiac compromise in the patient. She requires continued cardiology and neurology follow-up for management of mitral insufficiency and chorea, respectively.

## 3. Discussion

The incidence of ARF and RHD is largely unknown in the United States due to widespread access to early detection and treatment of GAS but is thought to be ≤2 cases per 100,000 school-aged children [5]. Our case of ARF is singular in its presentation with concurrent COVID-19 infection. Surprisingly, our patient described neither streptococcal symptoms (e.g., pharyngitis and impetigo) nor COVID-19 symptoms such as fever, respiratory symptoms, or systemic signs of viral infection. Instead, she presented with isolated choreiform movements, which is the most rare presentation of ARF.

Despite adequate treatment of ARF with antibiotics, this patient continues to exhibit chronic fatigue and mild choreiform movements several months later. Importantly, this patient's ejection fraction is normal and RHD remained stable throughout her disease course, making cardiogenic causes of fatigue unlikely. Subclinical hypothyroidism was considered as a cause of her fatigue given the patient's elevated TSH on presentation, but repeat thyroid function tests returned normal. TSH is an acute phase reactant and frequently elevated secondary to inflammation, as was likely the case in this patient. Finally, her ongoing fatigue may be due to residual ARF symptoms; however, her coinfection with COVID-19 suggests an alternative pathological etiology exists. Fatigue following COVID-19 infection has been documented in a significant proportion of patients 60–90 days after acute disease [6].

It is unclear what, if any, synergistic role COVID-19 may have with GAS that predisposed our patient to develop ARF. Multiple reports document a clear correlation between bacterial superinfections and viral respiratory infection, with a similar phenomenon occurring in COVID-19 [3]. Increased susceptibility to bacterial infection in the setting of viral illness occurs through several mechanisms, including dysregulation of mucociliary clearance, resulting in increased colonization by bacteria. Cardiac involvement is a major diagnostic criteria for ARF, and our patient's mitral valve involvement is a classic finding. COVID-19 can also cause myocarditis and valvular insufficiency [7]. The constellation of symptoms and multisystem involvement begs the issue of whether *SARS-CoV-2* infection potentiates an individual to rheumatic fever or vice versa.

In addition to cardiovascular changes, neurologic involvement is prevalent in both COVID-19 and ARF. Sydenham's chorea is a well-documented component of ARF, comprising one of the major diagnostic JONES criteria. Poststreptococcal movement disorders also exist, such as in pediatric autoimmune neuropsychiatric disorders associated with streptococcal throat infections (PANDAS) [8]. While PANDAS was considered for this patient, ARF was felt to be more consistent with her presentation. There have been numerous reports of neurological manifestations during and after COVID-19 infection including altered mental status, neuropsychiatric illnesses, and cerebrovascular events [9].

Indeed, the list of associated symptoms and potential long-term consequences of COVID-19 infection continues to expand as we learn more about this deadly disease. We have presented here a case report of a well-studied and familiar disease in the context of a COVID-19-positive pediatric patient. We have also provided a discussion of mechanisms whereupon these two diseases may overlap or synergize, knowing full well that our discussion points are limited due to the nature of a case report. We anticipate that the dissemination of this knowledge will aid other clinicians and educate the medical community regarding the subtle ways that COVID-19 infection and ARF can present.

## Data Availability

All data used to support the findings of this study are available upon request to the corresponding author.
